# Pandora's Box

**DOI:** 10.1192/bji.2022.8

**Published:** 2022-05

## Overdosing, race and the pandemic

The COVID pandemic has highlighted widespread existing health inequalities in the UK and elsewhere. A research letter just published in *JAMA Psychiatry* reveals that drug overdose mortality rates have increased sharply in the USA since the pandemic started, in 2020. Using data from the Centers for Disease Control and Prevention (Wide-ranging Data for Epidemiologic Research) and the National Center for Health Statistics, they calculated drug overdose death rates by race and ethnicity for 1999–2020 in the USA.

They found that drug overdose mortality rates had been rising since 2015 most rapidly among ‘Black, Hispanic and Latino’ communities. In 2020 the largest percentage increase (48.8%) was in Black individuals compared with White individuals (26.3%). They also report that in American Indian and Alaskan natives, who between 1999 and 2017 had the same overdose mortality as White individuals, the overdose mortality rates climbed in 2019 (at 28.9 per 100 000 compared with 25 for the White population) and became the highest (41.4 per 100 000) in 2020; that was 30.8% higher than the rate increase in White individuals.

The authors discuss the role of illicit drug use and toxic polysubstance availability of synthetic opioids, benzodiazepines and highly purified methamphetamines as contributing to the worsening overdose crisis in the USA. They observe that the high and unpredictable potency of illicit drug supply may be disproportionately harming racial and ethnic minority groups with deep-seated inequalities in social conditions (housing, employment, policing and arrests, preventive care, harm reduction, telehealth, medications for opioid use disorder and naloxone access) playing a major part.

They conclude that overdose mortality has increased since the COVID-19 pandemic and they believe that their records from 2020, which were provisional, may be underestimating the mortality rates. They observe that overdose mortality is ‘increasingly becoming a racial justice issue in the US’ and ask for the social/health inequalities to be addressed.

Friedman JR, Hansen H. Evaluation of increases in drug overdose mortality rates in the US by race and ethnicity before and during the COVID-19 pandemic. *JAMA Psychiatry* [Epub ahead of print] 2 Mar 2022. Available from: 10.1001/jamapsychiatry.2022.000.PMC889236035234815

## Growing old and lost for words?

Despite our richer experience and wide-ranging vocabulary accumulated over the years, as we grow old, we often find ourselves unable to find the right word at the right time. Frustrating as it is, our brain refuses to play ball. What is the problem?

Investigators from the Max Plank Institute for Human Cognitive and Brain Sciences and Leipzig University investigated the underlying brain mechanisms involved in this. They compared a group of younger people aged 20–35 and older people aged 60–70 years. The participants were asked to name words in different categories (animals, vehicles metals, other) while undergoing magnetic resonance imaging (MRI) scanning. Although both age groups were able to find words, the older people were slower compared with the younger ones. This difference in performance, the researchers found, was down to functional brain network connectivity. More specifically, the younger people's brain activity showed more intensive exchange between the network where factual knowledge is stored (semantic memory) and the network responsible for general functions such as attention and memory (executive network) compared with the older group. In the latter, there was more intensive activity within the executive network, indicating more difficulty in these subjects, whereas the connectivity between the two networks was less effective.

Don't despair, as there are some things you can do to help yourself – exercise, sleep well and stay away from bugs that may get up your nose. For more info on these, please read the following three summary reports.

Martin S, Saur D, Hartwigsen G. Age-dependent contribution of domain-general networks to semantic cognition. Cereb Cortex 2022; 32(4): 870–90.3446444210.1093/cercor/bhab252PMC8841593

## Exercise so as to remember

As we age and our memory starts failing, we grasp at every piece of advice on how to stop or at least slow this down. Exercise figures high on the list, and Pandora has repeatedly reported on research demonstrating the merits of exercise on both body and mind. A lot has been said about aerobic exercise being one of the most promising approaches for enhancing cognitive function in mature age. Different agencies and apps offer advice on numbers of steps or miles to walk per day, some of us opting for the minimum requirements and others enthusiastically exceeding these. But does aerobic exercise improve memory?

A recently published systematic review and meta-analysis tried to clarify matters. They selected 36 randomised control trials with data from 2750 participants without dementia to determine whether aerobic exercise influences episodic memory (concerning remembrance of past personal events and experiences) in late adulthood (mean age 70 years) and what characteristics of the subjects and interventions may influence the effects. They found that aerobic exercise did indeed improve episodic memory in those with a mean age between 55 and 68 (young-old) but not those between 69 and 85 (old-old). However, where women were overrepresented (65–100%) in the older group, more benefit was noted from aerobic exercise.

If you want to know how much aerobic exercise you should do, based on their data, they advised that sessions lasting 15–90 min three times a week for 18–39 weeks, to achieve a total of >3900 min, were effective. Make sure you start early, and don't wait until you get into the old-old age, particularly if you are a man!

Aghjayan SL, Bournias T, Kang C, Zhou X, Stillman CM, Donofry SD,  Aerobic exercise improves episodic memory in late adulthood: a systematic review and meta-analysis. Commun Med 2022; 2: 15.10.1038/s43856-022-00079-7PMC905329135603310

## Sleep gets your brain cleaned

There is a lot we still don't understand about the physiology of the need to sleep, but we do know that one important function of sleep is to clear our brain from waste generated through our daytime activity. We also know that the cerebrospinal fluid, driven by cardiovascular, respiratory and vasomotor brain pulsations, flows along perivascular spaces and washes away our brain waste material.

A recent study from Norway investigated how these pulsations may change during sleep. In a group of 15 healthy volunteers (nine males and six females, with an average age of 26.5 years), they recorded simultaneously fast functional MRI, magnetic resonance encephalography (MPEG) and electroencephalography (EEG) during sleep and while awake. They quantified sleep-related changes in the signal frequency and also the strength of the brain pulsations.

They found that in non-rapid eye movement sleep, signal frequency was reduced whereas brain pulsation was increased compared with wakefulness. EEG slow oscillation power (delta waves) was increased in regions of the brain that showed sleep-related changes in MPEG pulsation. The increase in respiratory pulsation was greatest in the areas of the brain most used during the day, i.e. the visual cortex, auditory cortex and sensorimotor cortex, which needed more cleaning up during sleep.

They concluded that their results support the view that sleep promotes fluid transport in the brain via an increase in brain pulsation and waste clearance. They aim to investigate these functions in brain disorders associated with sleep disturbances such as Alzheimer's dementia and others.

Helakari H, Korhonen V, Holst SC, Piispala J, Kallio M, Väyrynen T,  Human NREM sleep promotes brain-wide vasomotor and respiratory pulsations. *J Neurosci* [Epub ahead of print] 7 Feb 2022. Available from: 10.1523/JNEUROSCI.0934-21.2022.PMC894423035135852

## Keep your nose clean

As Alzheimer's dementia is increasing in prevalence, intensive research into its causes is searching in all possible directions. A recent study discovered an unusual or at least not previously thought of culprit, *Chlamydia pneumoniae*, a pathogen that can take residence in the nose. The researchers isolated live organisms from tissues and using immunohistochemical methods showed that this pathogen can, via the olfactory and trigeminal nerves, reach the olfactory bulb and brain in mice within 72 h. Importantly they also showed that this resulted in dysregulation of key pathways known to be involved in Alzheimer's disease at 7 and 28 days following inoculation. They also detected amyloid beta accumulations close to the *C. pneumoniae* inclusions, at least in the olfactory system, and *in vitro* showed the pathogen to infect central nervous system glia.

They concluded that *C. pneumoniae* could invade the brain via the olfactory and trigeminal nerves and survive in glia, causing amyloid beta deposition.

Chacko A, Delbaz A, Walkden H, Basu S, Armitage CW, Eindorf T,  Chlamydia pneumoniae can infect the central nervous system via the olfactory and trigeminal nerves and contributes to Alzheimer's disease risk. Sci Rep 2022; 12: 2759.3517775810.1038/s41598-022-06749-9PMC8854390

## The haunted house experience

In the quest to understand anxiety, the body responses to fear have been widely investigated. However, practical and ethical considerations limit the scope of such investigations in terms of the threat used in experiments and the physical measures of the body response. A recent study from the Division of Humanities and Social Sciences at the California Institute of Technology aimed to examine scare responses in a more comprehensive way, incorporating a social component with the psychological and using a hitherto not considered type of threat experience.

They recruited 156 individuals, whom they divided into small groups, and exposed them to a 30 min haunted house experience. The house had 17 rooms, and the subjects went through them with a continuous exposure to threatening experiences such as mimicking suffocation threats, a speeding car and a shower of shots from a firing squad. During the experience in the haunted house, they wore monitoring wristbands to measure their electrodermal activity, sweat-induced changes in the skin's electrical characteristics, including skin conductance level and skin conductance response. Prior to entering the haunted house, the participants rated their expected fear on a scale from 1 to 10; they also rated their actual fear after the visit.

The researchers examined four factors from these data: (a) group composition; (b) threat imminence; (c) intrapersonal factors of fear; and (d) the participant's sensitivity to threats. Interestingly, they found positive associations between friends and tonic arousal, i.e. being around friends as opposed to strangers increased the arousal of being scared. There was also a positive association between unexpected threats and subjective fear. Those who felt the most frightened in the haunted house had more rapid changes to their body experiences. Individuals who had strong responses to the first room of the haunted house showed increased responses as they went through the other rooms.

A helpful take away message is, if you don't enjoy being scared, make sure that the next time you happen to visit a haunted house, you don't go with a friend!

Tashjian SM, Fedrigo V, Molapour T, Mobbs D, Camerer CF. Physiological responses to a haunted-house threat experience: distinct tonic and phasic effects. Psychol Sci 2022; 33(2): 236–48.3500171010.1177/09567976211032231PMC9096462

## Scared of death?

Do you wonder what you'll experience in those last moments of life? ‘Near death’ experience has been described by those surviving in various ways, including being outside their bodies, floating in space above and watching themselves being resuscitated. But what the dying brain experiences in the moments prior to final death is not known. Research in rats showed that the dying mammalian brain paradoxically generates neural correlates of heightened conscious processing.

An idea of what this is like in humans was unexpectedly observed by doctors at the University of Tartu, Estonia, as they were monitoring an 87-year-old patient, who was having epileptic seizures, with continuous EEG. The patient unfortunately suffered a heart attack and died while still being recorded with EEG. In order to investigate what happened in the 30 s before and after the heart stopping, they examined the EEG recording and measured 900 s of brain activity during that time. They saw changes in neural oscillations (brain waves), specifically in gamma but also in theta, delta, alpha and beta oscillations, which are involved in high cognitive functions such as dreaming, memory retrieval and conscious perception, like those associated with memory flashbacks. After cardiac arrest, all oscillations were decreased but higher gamma power was observed compared with that in the interictal interval prior to the cardiac arrest. There was modulation of left hemisphere gamma activity by alpha and theta rhythms across all windows, even after cessation of cerebral blood flow.

This is the first evidence from the dying human brain in a real life, non-experimental, clinical setting that demonstrates that the human brain may possess the capability to generate coordinated activity during the dying period. The authors comment ‘these findings challenge our understanding of when exactly life ends and generate important questions such as those related to the timing of organ donation’. This raises serious ethical questions, and more research is needed to establish more clearly when exactly life ends.

Vicente R, Rizzuto M, Sarica C, Yamamoto K, Sadr M, Khajuria T,  Enhanced interplay of neuronal coherence and coupling in the dying human brain. Front Aging Neurosci 2022; 14: 813531.3527349010.3389/fnagi.2022.813531PMC8902637

